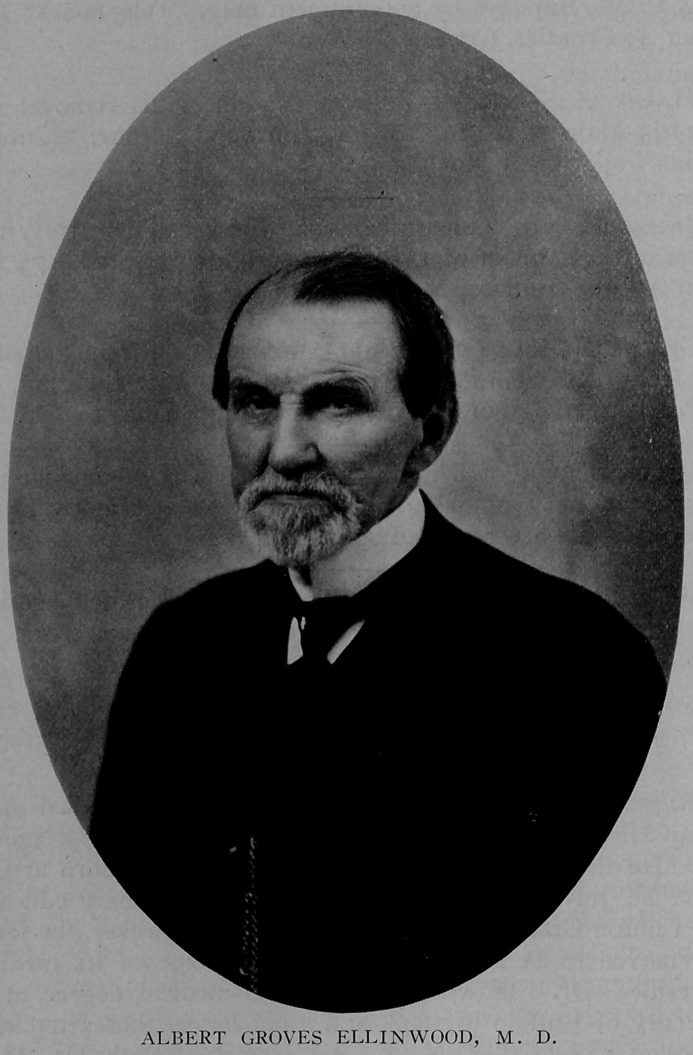# Dr. Albert Groves Ellinwood

**Published:** 1909-05

**Authors:** 


### OBITUARY

Dr. Albert Groves Ellinwood, of Attica, N. Y., died at the German Deaconess's Hospital, Buffalo, March 26, 1909, aged 84 years. He was the son of Eli Ellinwood, and was born at Clinton, N. Y., July 1, 1824. He received his preliminary education at the Clinton Grammar School. Meanwhile, however, the family had removed to Pembroke, N. Y., where some of its members still reside. Dr. Ellinwood received his medical degree at the University of Buffalo in 1848, and immediately thereafter began practice at Cowlesville, N. Y., in association with Dr. M. E. Potter. Later he removed to East Pembroke, and finally, in 1862, located at Attica where he continued to reside until his death.

Dr. Ellinwood enjoyed the confidence of a large clientele during his entire medical career. In 1855, he married Arlotta

Maria Bass, of Randolph, Mass., who died a few years ago. One son, Dr. F. F. Ellinwood, of Utica, and two daughters, both of whom married clergymen, survive.

Dr. Ellinwood kept his activity until about a month previous to his death. He was a member of the local and general medical societies, and served as surgeon of the Erie railway for some-

ALBERT GROVES ELLINWOOD, M. D.

thing more than forty years. He was a courtly gentleman, respected of all who knew him.

## Figures and Tables

**Figure f1:**